# The effect of combination therapy of allicin and fenofibrate on high fat diet-induced vascular endothelium dysfunction and liver damage in rats

**DOI:** 10.1186/1476-511X-9-131

**Published:** 2010-11-14

**Authors:** Weihong Li, Daxin Wang, Guohua Song, Chunxia Zuo, Xianfu Qiao, Shucun Qin

**Affiliations:** 1 Institute of Atherosclerosis and Department of Basic Medicine, Taishan Medical University, Taian, Shandong 271000, China; 2 Medical School, Yangzhou University, Yangzhou, Jiangsu 225001, China

## Abstract

**Background:**

It is designed to investigate the effects of combination therapy of allicin and fenofibrate on the endothelial and liver functions in rats with hyperlipidemia.

**Methods:**

The healthy male Wistar rats fed high fat diet were treated with fenofibrate (80 mg/kg per day) alone, allicin (60 mg/kg per day) alone and a lower dasage of combined therapy (allicin 20 mg/kg per day and fenofibrate 30 mg/kg per day) respectively for 8 weeks. The serum levels of cholesterol, triglyceride, nitrogen oxidative, alanine transferase (ALT) and aspartate transferase (AST) were determined. Acetylcholine-induced endothelium-dependent vascular relaxation (EDVR) of aorta rings was tested, and the morphologic changes of liver tissue were observed.

**Results:**

Compared with high fat diet control, fenofibrate alone or the combined therapy increased remarkably the levels of high density lipoprotein respectively (P < 0.05). Both single and combined therapy of fenofibrate and allicin significantly enhanced the levels of NO (P < 0.01 or P < 0.05), but the combined therapy had greatest high EDVR responses (P < 0.01). Furthermore, the reduced levels of ALT and AST were significantly obvious in the combined therapy groups (P < 0.01 or P < 0.05). In addition, the lower dosage of combined therapy significantly ameliorated severe fatty degeneration of liver cells occurred in the high fat diet fed rat although the single fenofibrate treatment showed spotty necrosis of liver cells and bile duct expansion.

**Conclusion:**

Combination therapy with allicin and fenofibrate can effectively enhance the protective effects on endothelial function and reduce the hepatic damage in rats with hyperlipidemia.

## Background

Dyslipidemia and subsequent atherosclerosis are recognized as the most dangerous inducers of ischemic cardiovascular disease (ICD) at present. Numerous research results demonstrated that the damage of vascular endothelial function (DVEF) as the early detectable alteration of ICD occurred before the appearance of typical evidences of ICD [1]. Therefore, the prevention and treatment of DVEF have a significant implication for the hyperlipidemic patients. Several researches indicated that fenofibrate as an important lipid-regulating drug could ameliorate vascular endothelial function (VEF) as well [2,3]. However, a long-term and high-dose application of fenofibrate could induce the possibility of liver function damage (LFD) [4,5]. In order to regulate blood lipid effectively, protect VEF and avoid the occurrence of drug-induced liver injury at the maximum degree, we investigated the protective effect of combination of allicin and fenofibrate on VEF as well as the combination influence on liver function in hyperlipidemic rats to provide definite theoretical and experimental basis for the clinical medication.

## Methods

### Animal and Feed

SPF Wistar rat (220~250 g, 4 months old) were provided by the laboratory animal center of Shandong Lukang Pharmaceutical Group (Animal Certification No.: SCXK LU 20080002). Chow diet was the standard feed offered by the laboratory animal center of Taishan Medical University, and the high-fat diet was consisted of cholesterol 2.0%, sodium cholate 0.5%, lard 10.0%, propylthiouracil 0.2%, white sugar 5.0% and basal feed 82.3% (corn flour 36%, wheat flour 35%, wheat bran 15%, soybean flour 10%, yeast powder 1%, salt 1%, bone powder 1% and fish liver oil 1%). The animal treatment was strictly carried out according to the guide lines of the laboratory animal ethical committee.

### Drug and Reagent

Allicin soft capsules were obtained from Xinjiang Xinbei Pharmaceutical Co., Ltd., and the lot number was 080911. Fenofibrate capsules used were from Laboratoires Fournier S. A., and the lot number was H20050004. Cholesterol and sodium cholate (analytical pure) were purchased from Beijing Solarbio Science & Technology Co., Ltd. and Shanghai Lanji Technology Development Co., Ltd., respectively. Propylthiouracil was the product of Shanghai Zhaohui Pharmaceutical Co. Ltd., and the lot number was 081206. Sodium pentobarbital was obtained from Sinopharm Chemical Reagent Shanghai Co., Ltd. (Imported and load separately). Phenylephrine (PHE) and acetylcholine chloride (Ach) were purchased from Sigma. Assay kits for total cholesterol (TC), triglyceride (TG), low-density lipoprotein cholesterol (LDL-C) and high-density lipoprotein cholesterol (HDL-C) used were from Zhejiang Elikan Biological Technology Co., Ltd. Assay kit for nitric oxide (NO) was obtained from Nanjing Jiancheng Bioengineering Institute. Assay kits for alanine transferase (ALT) and aspartate transferase (AST) were provided by Shanghai Kehua Dongling Diagnostic Products Co., Ltd. All the other reagents used were of the analytical pure.

### Major apparatus

Out body tissue organ constant temperature perfusion system (Chengdu Technology & Market Co., Ltd.), TL-18 M High speed tabletop refrigerated centrifuge (Shanghai Centrifuge Institute Co., Ltd.), 7600-020 Automatic bio-chemical analysis instrument (Hitachi, Ltd., Japan), 755B UV Visible spectrophotometer (Shanghai Precision & Scientific Instrument Co., Ltd.), 820 Microtome (AO Co., America), ZP-1 Film launching apparatus (Tianjin Tianli Aivation Electro-mechanical Co., Ltd.), Microscope and BX51 Micro-camera system (Olympus Corporation).

### Animal Modeling and Grouping

Sixty male Wistar rats were randomly allocated into normal control group, model group, fenofibrate group, allicin group, and combination group, each group consisted of 12 rats. The animals were acclimated for one week prior to duplication of hyperlipemia model for 4 weeks, and then drug intervention for 8 weeks after the model was established successfully. Normal control group was fed with general diet, model control and fenofibrate group were fed with high-fat diet, whilst allicin was added to the high-fat diet for allicin group and combination group at a dose of 60 mg/kg·d and 20 mg/kg·d, respectively. Fenofibrate group and combination group were orally administrated with fenofibrate at a dose of 80 mg/kg and 30 mg/kg once a day, respectively. Normal control group, model group and allicin group were administrated with the same volume of distilled water orally once a day. After consecutive drug intervention for 8 weeks, till the end of the 12 weeks' experiment, one rat died in normal control group, three rats died in model group, and two rats died in each of the rest three groups.

### Observation Indexes

Blood was sampled by tail-cutting method at the end of the fourth week for blood lipids measurement to confirm successful establishment of the hyperlipemia model. The rats were anaesthetized by injection of sodium pentobarbital 50 mg/kg, and then were executed after abdominal aorta blood sampling, and subsequently, the blood lipid, serum NO, ALT and AST levels were detected. Thoracic aortas from the rats were used to prepare thoracic aorta rings for isolated relaxation effect measurement, and the liver tissue samples were prepared for histomorphological examination. (1) Measurement of Blood Lipid: Serum TC and TG were determined according to liquid double-reagent enzymatic method, and HDL-C and LDL-C were assessed by direct method on a Hitachi 7600-020 automatic bio-chemical analysis instrument. (2) Measurement of serum NO: Nitric acid reductase method. (3) Measurement of Serum ALT and AST: Serum ALT and AST levels were detected by UV-lactate dehydrogenase method and UV-malate dehydrogenase method, respectively, on a Hitachi 7600-020 automatic bio-chemical analysis instrument. (4) Function measurement of endothelium-dependent vascular relaxation (EDVR) [6]: After abdominal aorta blood sampling, the anaesthetized rats by injection of sodium pentobarbital 50 mg/kg were subjected to thoracotomy rapidly, the isolated thoracic aortas were kept at 37 °C Krebs-Henseleit (K-H) solution with persistent airing commixed gas composed of 95% Oxygen and 5% carbon dioxide. The thoracic aorta from each rat was made into thoracic aorta rings with a length of 3 mm by means of dissecting microscope, the excellent without damage rings were carefully moved to a thermostatic water bath containing 37 °C K-H solution with persistent airing the above commixed gas, the downside of the rings were immobilized to the bottom of the thermostatic bath by steel-hooks, while their upside was connected to BL-420 biological and functional experimental system through tension transducers, the rest tension was adjusted to 1.5 g (tested to be the optimum tension by pre-experiment) and balanced for 30 minutes. And then, potassium chloride solution (with a final concentration of 50 mmol/L in the bath solution) was added to give pre-stimulation for two times. After tension balance, PHE with a final concentration of 1 × 10^-6 ^mol/L was added to the thermostatic bath to make the thoracic aorta rings have a pre-contraction, and once the contraction of the rings reached the platform stage, a sequence increasing concentration of Ach (accumulated concentration) with a final concentration of 1 × 10^-8^mol/L, 5 × 10^-8^mol/L, 1 × 10^-7^mol/L...... and 5 × 10^-4^mol/L, respectively, was used for inducing vasodilation, and the induced tension alterations of the thoracic aorta rings were recorded synchronously. Using relaxation rate induced by PHE to express the relaxation effect induced by Ach under different accumulated concentrations, and thus, Relaxation Degree = (tension of the ring induced by PHE - tension of the ring induced by Ach) ÷tension of the ring induced by PHE × 100%. (5) Liver histomorphological examination: The isolated fresh liver tissues were immediately fixed with paraformaldehyde for 24 h, and after ethanol dehydration and regular paraffin embedding, they were made into 5 μm paraffin slices, and finally applied to HE staining.

### Statistical analysis

All the data were expressed as means ± standard deviation (SD). The measurement data of multiple groups were compared with one-way ANOVA, the comparison between two groups was performed with (LSD) T-Test, and a value of P < 0.05 was considered significant.

## Results

### Hyperlipemia Rat Model Induced by High-fat Diet

Four weeks feed resulted in significant increase of serum TC, TG, LDL-C and HDL-C levels (P < 0.01) in high-fat diet groups compared to normal control group (Table 1). However, there was statistically non-significant within groups of high-fat diet.

**Table 1:** Serum lipids in rats before administration of medication

Groups	n	TC(mmol/L)	TG(mmol/L)	HDL-C(mmol/L)	LDL-C(mmol/L)
Normal control	11	1.760 ± 0.305	0.501 ± 0.086	1.362 ± 0.143	0.245 ± 0.034
Model control	9	4. 929 ± 2.142 ^a^	0.922 ± 0.178^a^	1.578 ± 0.173 ^a^	2.428 ± 0.513 ^a^
Fenofibrate	10	5.153 ± 2.668 ^a^	0.908 ± 0.228 ^a^	1.662 ± 0.141 ^a^	2.370 ± 0.539 ^a^
Allicin	10	4. 893 ± 2.105^a^	0.948 ± 0.251 ^a^	1.626 ± 0.129^a^	2.499 ± 0.489 ^a^
Combination	10	5.007 ± 2.377 ^a^	0.949 ± 0.241 ^a^	1.590 ± 0.149 ^a^	2.421 ± 0.460 ^a^

TC: total cholesterol; TG: triglyceride; HDL-C: high-density lipoprotein cholesterol; LDL-C: low-density lipoprotein cholesterol; Values are means ± SD; a: Compared to Normal control, P < 0.01

### Influence of drug intervention on rat blood-lipid levels

As shown in Table 2, compared with the normal control group, serum TC, TG, LDL-C and HDL-C levels were increased dramatically (P < 0.01) in the other four groups. Serum TC, TG, LDL-C and HDL-C levels of the drug intervention groups decreased significantly (P < 0.01), while serum HDL-C levels of fenofibrate group and combination group enhanced remarkably (P < 0.05) compared to model control group. Serum LDL-C level of the allicin group also reduced but the lowering degree exceeded neither that of fenofibrate group (P < 0.01) nor that of combination group (P < 0.01) with a lower dosage. Furthermore, serum LDL-C level of combination group was slightly lower than the group treated with fenofibrate alone, but not reach statistical significant.

**Table 2:** Serum lipids and NO levels in rats at the end of 12 weeks treatment

Groups	n	TC(mmol/L)	TG(mmol/L)	HDL-C(mmol/L)	LDL-C(mmol/L)	NO(μmol/L)
Normal control	11	1.726 ± 0.199	0.519 ± 0.053	1.344 ± 0.109	0.252 ± 0.024	38.047 ± 3.604
Model control	9	10.592 ± 2.258 ^a^	1.789 ± 0.078^a^	1.722 ± 0.092 ^a^	6.272 ± 0.552 ^a^	23.649 ± 4.875 ^a^
Fenofibrate	10	4.853 ± 0.670 ^ac^	0.668 ± 0.061 ^ac^	1.832 ± 0.065 ^ad^	2.370 ± 0.263 ^ac^	32.729 ± 2.906^bc^
Allicin	10	5.183 ± 0.718^ac^	0.738 ± 0.071 ^ac^	1.793 ± 0.051^a^	3.049 ± 0.278 ^ace^	30.520 ± 3.194 ^ac^

Combination	10	4.307 ± 0.490 ^ac^	0.689 ± 0.060 ^ac^	1.829 ± 0.055 ^ad^	2.251 ± 0.367 ^ac^	33.019 ± 3.879^bc^

TC: total cholesterol; TG: triglyceride; HDL-C: high-density lipoprotein cholesterol; LDL-C: low-density lipoprotein cholesterol; NO: nitric oxide; Values are means ± SD; a and b: Compared to Normal control, a P < 0.01, bP < 0.05; c and d: Compared to Model control, cP < 0.01, dP < 0.05; e: Compared to Fenofibrate, e P < 0.01

### Influence of drug intervention on rat serum NO levels

Compared with the normal control group, serum NO levels of the rest groups were reduced markedly (P < 0.01 or P < 0.05). However, serum NO level of the allicin group increased and closed to that of fenofibrate group when comparing with model control group, additionally, combination group seemed to have the most significant increase, but showed no additive effect (Table 2).

### Influence of drug intervention on rat ALT and AST levels

Compared with normal control group, serum ALT and AST levels of the rest groups were all increased markedly (P < 0.01 or P < 0.05). Serum ALT and AST levels lowered significantly in the drug intervention groups(P < 0.01) when comparing with model control group. In addition, serum ALT and AST levels markedly reduced in allicin group and combination group compared to fenofibrate group (P < 0.01) (Table 3).

**Table 3:** Serum ALT and AST levels in rats at the end of 12 week treatment

Groups	n	ALT(U/L)	AST(U/L)
Normal control	11	35.364 ± 2.730	81.546 ± 5.087
Model control	9	70.333 ± 3.500 ^a^	134.778 ± 4.790 ^a^
Fenofibrate	10	49.200 ± 4.022 ^ac^	102.100 ± 5.840 ^ac^
Allicin	10	41.500 ± 3.375 ^acd^	92.100 ± 5.763 ^acd^
Combination	10	42.100 ± 3.665 ^acd^	90.400 ± 8.343^bcd^

ALT: alanine transferase; AST: aspartate transferase; Values are means ± SD; a and b: Compared to Normal control, ^a^P < 0.01,^b^P < 0.05; c: Compared to Model control, P < 0.01; d: Compared to Fenofibrate, P < 0.01

### Function Alteration of Thoracic aorta EDVR and Influence of Drug Intervention on Hyperlipemia Rat

Relaxation degrees of thoracic aorta rings of the model control group were decreased remarkably (P < 0.01) under each of the accumulated concentration of Ach compared to the normal control group. However, compared with the model control group, all of the relaxation degrees of thoracic aorta rings of the drug intervention groups significantly increased (P < 0.01) when the accumulated concentration of Ach reached 5 × 10^-8 ^mol/L. Furthermore, compared with single drug treatment groups, allicin group and fenofibrate group, relaxation degrees of thoracic aorta rings of combination group raised dramatically (P < 0.05) since the accumulated concentration of Ach reached 5 × 10^-5 ^mol/L, and which seemed to have additive effect (Table 4).

**Table 4:** Ach-induced vascular relaxation of aorta rings in rats at the end of 12 week treatment (%)

Ach (mol/L)	Normal control	Model control	Fenofibrate	Allicin	Combination
1 × 10^-8^	9.504 ± 3.361	4.894 ± 1.838^a^	7.924 ± 3.095	7.727 ± 2.524	8.160 ± 2.661
5 × 10^-8^	29.501 ± 5.825	10.468 ± 4.811^a^	19.967 ± 5.227^ac^	21.199 ± 5.952^bc^	24.344 ± 6.081^c^
1 × 10^-7^	45.167 ± 6.798	21.421 ± 4.423^a^	35.535 ± 5.401^ac^	37.791 ± 6.251^bc^	38.110 ± 4.599^bc^
5 × 10^-7^	59.172 ± 5.920	28.409 ± 5.214^a^	42.072 ± 6.291^ac^	43.341 ± 3.877^ac^	45.893 ± 5.357^ac^
1 × 10^-6^	65.610 ± 4.660	33.799 ± 5.341^a^	51.420 ± 5.638 ^ac^	50.197 ± 6.689 ^ac^	53.360 ± 5.938 ^ac^
5 × 10^-6^	78.351 ± 5.409	41.251 ± 4.535 ^a^	59.392 ± 5.384 ^ac^	57.945 ± 3.376 ^ac^	61.576 ± 3.600 ^ac^
1 × 10^-5^	84.039 ± 3.895	45.537 ± 4.862 ^a^	66.847 ± 5.710 ^ac^	64.017 ± 4.827 ^ac^	69.594 ± 4.474 ^ac^
5 × 10^-5^	86.903 ± 4.872	49.129 ± 5.366 ^a^	68.593 ± 4.449 ^ac^	67.532 ± 3.549 ^ac^	75.037 ± 5.364 ^acd^
1 × 10^-4^	89.075 ± 4.248	48.627 ± 4.031 ^a^	67.903 ± 4.853 ^ac^	67.182 ± 5.459 ^ac^	74.959 ± 4.344 ^acd^

Ach: Acetylcholine; Values are means ± SD; a and b: Compared to Normal control, ^a^P < 0.01, ^b^P < 0.05; c: Compared to Model control, P < 0.01; d: Compared to Fenofibrate, P < 0.05

### Liver histomorphological examination: Naked-eye observation

Livers of normal control group had regular shape with sharp edges, and red with glossy. Livers of model control group were hyperplastic with blunt edges, light yellow, and cut surface was oily to the touch. The naked-eye observation results of the rest three groups lied between the former two groups.

### Optical microscope inspection

**Figure 1:**
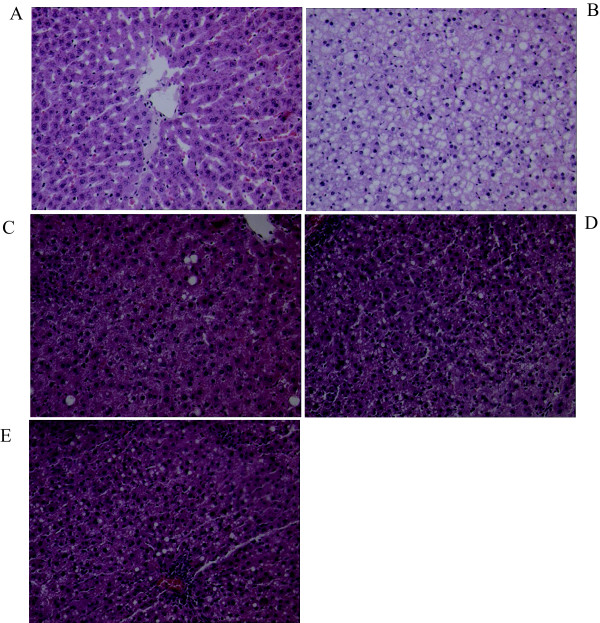
**The liver tissue HE Staining with 100 folds of amplification in rats at the end of 12 week treatment**. A: Normal control; B: Model control; C: Fenofibrate treatment; D: Allicin treatment; E: Combination treatment.

In normal control group, the hepatic lobules had clear structure, hepatocytes arranged orderly with cell nucleuses located centrally and without fatty denaturalization. However, in model control group, hepatic cord arranged in disorder, structure of hepatic lobules were damaged, with many lipid-droplets vacuoles in hepatocytes, which showed serious fatty denaturalization. In fenofibrate group, hepatocytes had mild fatty denaturalization with spotty necrosis and expanded bile ductule. The fatty denaturalization of hepatocytes decreased obviously in garlic group and combination group, and did not find spotty necrosis and expanded bile ductule in the latter group, furthermore, the disordered hepatocytes were restored to some extent in combination group (Figure 1).

## Discussion

Vascular endothelium as a widely distributed and an active metabolism organ (or system) with various bioactivity plays an important role in maintaining the stability of the vascular function, and the damage of VEF as the early detectable alteration of atherosclerosis is closely correlated with the occurrence and development of atherosclerosis [7]. The secretion decrease of endothelium derived relaxing factor (EDRF, that is NO) with vasodilative effect, is the most obvious phenomenon accompanied with the damage of VEF, therefore, serum NO level and function of thoracic aorta EDVR were measured to evaluate the VEF in this study.

This research showed that high-fat diet could induce hyperlipidemia in rats, and hyperlipidemia could decrease serum NO level and reduce function of EDVR significantly. The exact occurrence mechanism of damage of VEF induced by hyperlipidemia is not clearly understood, however, the endothelium derived NO is acknowledged to play a key role in regulating VEF. Some results indicated that LDL particles not only could enter the arterial walls easily but also be prone to oxidative modification, and the oxidized LDL have high cytotoxicity, which could directly damage vascular endothelium. Through inhibiting activity of nitric oxide synthase to reduce the production of NO or by inducing the high expression of adhesion molecules at the surface of the endothelial cells to promote inflammatory response, and finally lead to the damage of VEF [8].

It has been reported that 'high-fat diet' can induce lipid accumulation in rat liver by a nutritional intervention [9]. Other studies have revealed that high-fat diets promote hyperlipidemia [10] and hyperglycemia [11], and numerous researchers have examined their effects on muscle and liver physiology as well as endothelial function [12-14]. From this experience, it is generally accepted that high-fat diets can be used to generate a valid rodent model for the analysis of the pathophysiology of dyslipidemia [15-17]. Therefore, in this study, high fat diet-induced dyslipidemia rat model was used to examine the combined effects of allicin and fenofibrate on dietary induced lipoprotein changes and liver damage.

As a synthetic ligand of peroxisome proliferator-activated receptor α (PPARα), the main function of fenofibrate, a phenoxy aromatic acid lipid-lowering drug, is to reduce serum TG, but it also could decrease serum TC and LDL-C, increase HDL-C, and decrease the contents of LDL-C particles and apolipoprotein B (ApoB) to some extent [18]. Our present findings indicated that fenofibrate could ameliorate the abnormal blood lipid alteration in rats with hyperlipidemia as well as improve function of thoracic aorta EDVR, all of which were consistent with earlier studies that have demonstrated that fenofibrate could ameliorate blood stream mediated vasodilation in brachial artery in patients with hyperlipidemia [2,3]. The protective mechanism of fenofibrate on VEF may be related with other underlying factors except for its lipid regulating effect. Some results revealed that fenofibrate could increase the synthesis of NO through upregulating gene expression of endothelial NO synthase (eNOS) directly or by activating PPARα to inhibit signaling pathways associated with inflammatory response, and subsequently increase the activity of eNOS [19].

As a food material which can be used as medicine and food, garlic has been widely used as edible food for thousands of years in Asian countries such as China. Allicin, with the chemical name of diallyl thiosulfinate, and the major pharmacological component of garlic, has attracted attention of the international medical field gradually due to its therapeutic effects on various cardiovascular diseases. Results indicated that garlic extract could decrease serum TC and LDL-C levels markedly and increase HDL-C level in animals [20,21], and improve the serum TC and LDL-C levels significantly in clinical research [22]. Williams MJ et al. [23] have showed that garlic extract could ameliorate the damaged VEF in patients with coronary heart disease. Gonen A et al. [24] studied the anti-atherosclerosis mechanism of pure allicin in ApoE^-/-^/LDLR^-/- ^double-gene mutant mice, and results indicated that allicin could exert anti-atherosclerosis effects through lipoprotein modification or by inhibiting phagocytic uptake and degradation of LDL, except for antioxidant. This study showed that allicin could effectively decrease serum TC, TG and LDL-C levels in hyperlipidemia rats, while the HDL-C level did not change significantly, moreover, VEF gained protection, all of which were basically consisted with the previous reports. This report demonstrated for the first time that halving dosage combination of garlic and fenofibrate have an equivalent effect on lipid regulating effect in hyperlipidemia rats with that of double dosage of fenofibrate alone, and the former could improve protective effect on VEF compared with single usage of either garlic or fenofibrate. The research indicated that garlic and fenofibrate have synergistic effects on regulating blood lipid and improving VEF, additionally, the combination exist other underlying mechanism on ameliorating VEF except for that of lipid regulation.

Another important discovery of the research is that combination of garlic and fenofibrate could reduce damage of liver function as well as drug-induced liver injury. Fibrates (including fenofibrate) like most of the other presently applied clinical lipid regulating drugs have some serious toxic and side effects, such as the uprising of serum transaminase and creatine kinase, therefore, they are unsuitable for the patients with serious diseases associated with liver, gall bladder and kidney. Research in recent years indicated that application of fenofibrate at the dose of 300 mg/kg·d for 14 days, the liver function got serious damage except for the increase of β-oxidation of fatty acid and decrease of serum TC level, and some results showed that oxidative stress participated this process [4]. Some experiments in vitro indicated that high concentration fenofibrate could mediate death of human hepatoblastoma cells (HepG2) through enhancing reactive oxygen species or by inhibiting synthesis of glutathione, which confirmed that oxidative stress did participated in the cytotoxicity of fenofibrate [5]. Compared with single usage of fenofibrate with double dosage, halving dosage combination of garlic and fenofibrate could significantly alleviate damage of liver function, which may related with the dosage difference of fenofibrate on the one hand. In addition, garlic may antagonize the hepatotoxicity of fenofibrate to some extent when considering its antioxidant activity, however, the exact mechanism requires additional research.

## Conclusions

This study indicated that halving dosage combination of garlic and fenofibrate have an equivalent effect on lipid regulating effect in hyperlipidemia rats with that of double dosage of fenofibrate alone. Combination could improve protective effect on VEF, and reduce damage of liver function induced by hyperlipidemia as well as drug-induced liver injury, however, the underlying mechanism of combination on ameliorating VEF except for that of lipid regulation and the exact mechanism of alleviating drug-induced liver injury need to be further investigated.

## Competing interests

The authors declare that they have no competing interests.

## Authors' contributions

WL carried out the study design, data collection and analysis, the animal experiment and drafted the manuscript. DW was responsible for the funding and the design of the study. GS was involved in drafting the manuscript and revising it critically for important intellectual content. CZ participated in the animal modeling. XQ carried out the biochemical analysis. SQ was responsible for the study design, the funding, the data analysis, and the manuscript draft. All authors read and approved the final manuscript.
